# *Giardia lamblia* mimicking acute graft versus host disease after allogeneic hematopoietic stem cell transplantation: A case report

**DOI:** 10.1097/MD.0000000000021752

**Published:** 2020-08-14

**Authors:** Shengling Ma, Han Yan, Wei Shi, Yong You, Zhao-Dong Zhong, Yu Hu

**Affiliations:** Institute of Hematology, Union Hospital, Tongji Medical College, Huazhong University of Science And Technology, Wuhan, China.

**Keywords:** diarrhea, *giardia lambli*a, giardiasis, graft-versus-host disease, hematopoietic stem cell transplantation

## Abstract

**Rationale:**

As the major complications post allogeneic hematopoietic stem cell transplantation (allo-HSCT), gastrointestinal disorders were most commonly ascribed to acute graft-versus-host disease (aGVHD) and opportunistic infections. Though *Giardia lamblia* (*G lamblia*) is the most common waterborne parasite of intestinal infection worldwide, seldom has it been reported in a patient with acute severe aplastic anemia after allo-HSCT.

**Patient concerns:**

A 23-year-old male with severe aplastic anemia developed diarrhea, abdominal cramps, bloating, nausea, vomiting, fever, weight loss, and fatigue after allo-HSCT.

**Diagnosis:**

Stool examinations for ova and parasites showed *Giardia* trophozoites and cysts.

**Interventions:**

Methylprednisolone was stopped and the patient was intravenously treated with a 7-day course of metronidazole (500 mg, tid.). Simultaneously, cyclosporine (5 mg/kg) was continually utilized for suspicious gut GVHD.

**Outcomes:**

The *Giardia lamblia* in stool turned negative and his symptoms were resolved after the 7-day course.

**Lessons:**

Incorporating non-invasive monitoring of stool examination for ova and parasites in the follow-up algorithm for post-HSCT patients can expedite clinical decision-making in the differential diagnoses for aGVHD even in the non-endemic area. Metronidazole therapy can be well-tolerated in HSCT patients with giardiasis.

## 1 Introduction

As the major complications post allogeneic hematopoietic stem cell transplantation (allo-HSCT) within 100 days, gastrointestinal (GI) disorders were most commonly ascribed to acute graft-versus-host disease (aGVHD), medications, bacterial, viral, or fungus infections, and so on^[1]^ but some rare etiologic factors are always neglected.

*Giardia lamblia* (*G lamblia*), an intestinal protozoan parasite, is distributed worldwide and estimated to cause 280 million GI infections annually.^[2,3]^ The similar clinical profiles to those of aGVHD hamper the consideration of this rare differential diagnosis.^[1,4]^ Consequently, negligence of detection can result in misdiagnosis, introducing the strengthening of immunosuppression for its treatment, which can favor, accelerate, and/or aggravate *G lamblia* infection or even be life-threatening. Thus, data about giardiasis following HSCT are very limited. Herein, we present a patient with manifestations consistent with aGVHD after allo-HSCT where further work-up unexpectedly revealed intestinal infection of giardiasis.

## 2 Case report

A 23-year-old man with acute severe aplastic anemia was allografted with 15.55 × 10^8^ nucleated cells and 9.56 × 10^6^ CD34-positive cells from a 5/10-HLA-matched related donor after conditioning with busulfan 6.4 mg/kg and cyclophosphamide 200 mg/kg. Prophylaxis for GVHD consisted of Cyclosporin A, short-term methotrexate, and anti-thymocyte globulin 10 mg/kg. Ruxolitinib and mycophenolate mofetil were used as immunosuppressive therapy afterward. He also received trimethoprim-sulfamethoxazole, acyclovir and voriconazole for continuous infection prophylaxis. Engraftments of neutrophils and platelets were documented on day +9 and day +8, respectively.

From day +44, the patient reported loose stools twice a day with fever (99–100.4°F). With regard to the foregoing rash (Grade I°), elevated liver enzyme and bloating, methylprednisolone (40 mg per 12 hours) and cyclosporine (5 mg/kg) were utilized for initial treatment of suspected aGVHD. He was discharged on +67 when symptoms were alleviated.

**Figure 1:**
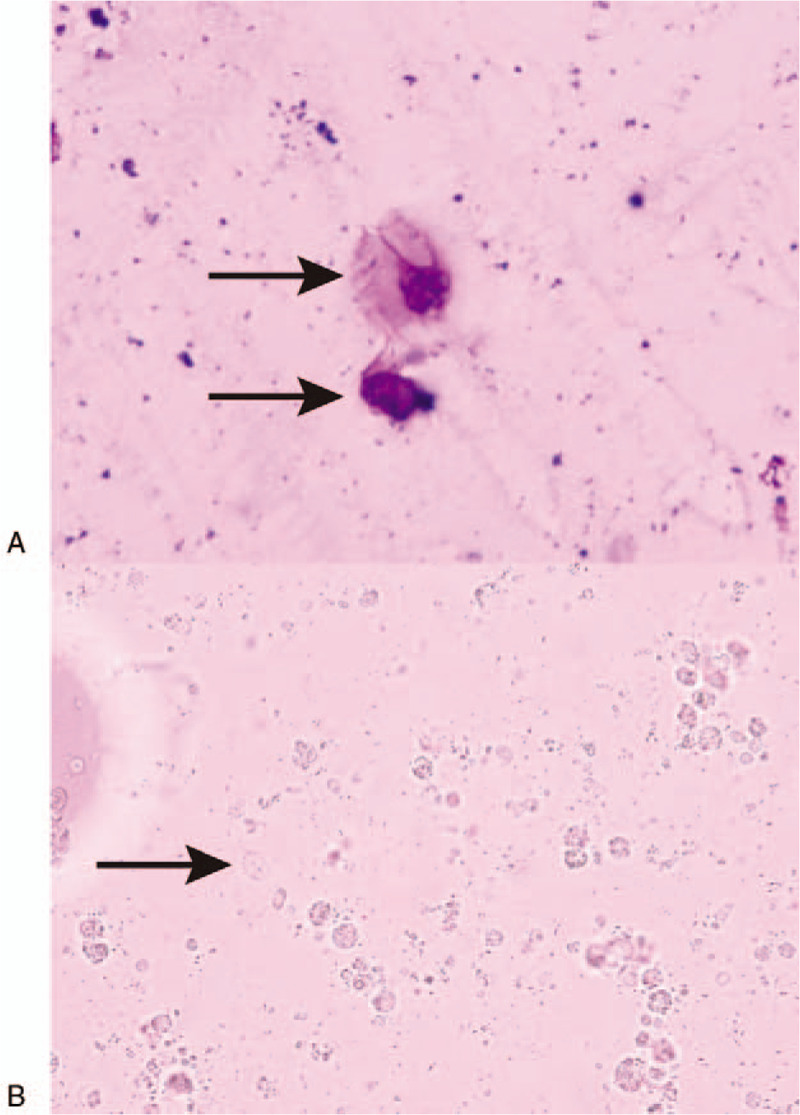
Three consecutive microscopic examinations of the patient's fresh fecal specimens all showed trophozoites and cysts of *Giardia lamblia* (arrow); (A) Wright staining, magnification 40×; (B) Saline, magnification 10×.

However, on day +69, the patient was re-admitted for worsen GI symptoms --green-watery diarrhea, abdominal cramps, bloating, nausea, vomiting, fever (99–100.4°F) and fatigue. The frequency and the amount of defecation increased to more than 10 times per day with a total volume of 1000 ml. His laboratory results were as follows: white blood cell count 6.61G/L (neutrophils 51.8%, lymphocytes 32.5%, monocytes 8.0%, and eosinophil 7.0%), hemoglobin 120 g/L, platelets 396 G/L, C-reactive protein 155.3 mg/L. Renal and hepatic function tests showed normal levels. The patient tested negative for cytomegalovirus and Epstein-Barr virus, human immunodeficiency virus, syphilis, and hepatitis B virus. Additionally, he lost around 10 kg over the past 2 months. He was initially suspected of aGVHD according to his former symptoms after HSCT. Nevertheless, on the first day of hospitalization, stool examination for ova and parasites showed *Giardia* trophozoites and cysts (Fig. 1, repeated 3 times). Upon further questioning, he revealed the consumption of relatively unsanitary food after HSCT.

**Figure 2:**
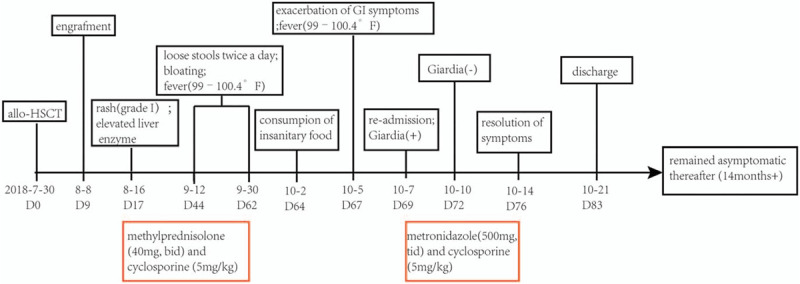
Timeline of the major clinical events. Allo-HSCT = allogeneic hematopoietic stem cell transplantation; GI = gastrointestinal.

Therefore, methylprednisolone was stopped and the patient was intravenously treated with a 7-day course of metronidazole (500 mg, tid.) which was well-tolerated. Notably, after treatment for only 2 days, *G lamblia* and white blood cells in stool turned negative, while red blood cells diminished promptly. Meanwhile, his GI symptoms remarkably alleviated during the treatment course. After being discharged from the hospital on day +83, the patient remained asymptomatic during further follow-up. (The timeline in Fig. 2 summarized the main clinical events after allo-HSCT). The patient has provided informed consent for publication of the case.

## 3 Discussion

*G lamblia* is a non-invasive intestinal flagellated protozoan parasite of the upper small intestine. As the most common waterborne parasitic infection of the human intestine worldwide, giardiasis was included in the World Health Organisation's Neglected Disease Initiative since 2004.^[5]^ Via consumption of cysts in contaminated food and water, *giardiasis* can be asymptomatic or symptomatic characterized by watery diarrhea, nausea, epigastric pain, vomiting, and weight loss. These symptoms appear 6 to 15 days after infection.^[6]^ While extensive data are available in children,^[7]^ people with human immunodeficiency virus^[8]^ and solid organ transplant (renal transplant,^[9,10]^ kidney or liver transplant,^[11]^ heart transplant,^[12,13]^ intestinal transplant,^[14]^ pancreas-kidney transplantation),^[15]^ rarely has giardiasis been reported post allo-HSCT in detail. According to our literature review of relevant studies, it can occur either in autologous HSCT^[16]^ or allo-HSCT,^[1,18]^ children^[16,18]^ or adults,^[17]^ peri-transplantation period^[19]^ or post-transplantation,^[19,20]^ asymptomatic^[20]^ or symptomatic.^[16,17,19]^

The origin of the infection in the patient reported here might be the consumption of contaminated food. Furthermore, we considered it possible that his significant weight loss, malabsorption, mild GI symptoms as well as fever in the second-month after HSCT might be the subacute stage of infection.

Giardia may be detected by microscopy, immunologic, or molecular methods.^[21]^ A single microscopic examination has a sensitivity of 35% to 50%, but it increases up to 70% to 90% after testing more samples of the same patient.^[22]^ Immunological tests differ in sensitivity and specificity, reaching up to 99%. Enzyme immunoassay test enzyme immunoassay identifies the *Giardia* antigen; cysts may be detected using direct immunofluorescence techniques.^[23]^ Verification, if needed, is performed through duodenal biopsy and aspiration. Because of invasiveness, the aforementioned method is used only in case of chronic GI symptoms without parasites detected in a stool sample.^[24]^ The timely diagnosis in our case has important clinical implications that incorporating non-invasive monitoring of stool examination for ova and parasites in the follow-up algorithm for post-HSCT patients can expedite clinical decision-making in the differential diagnoses for aGVHD.

Though there is no guideline on how patients with *Giardia i*n this special case of allogeneic HSCT should be treated, the patient was successfully treated with metronidazole in the classical way.^[25]^ Meanwhile, it is important not to neglect the management for presumptive gut GVHD since GVHD can represent both a source of misdiagnosis and a potential trigger for infectious diseases.

Our report highlights the fact that giardiasis can mimic aGVHD for GI disorders post allo-HSCT and should be part of the differential diagnosis even in the non-endemic area, underlining the importance of good hygiene, hand washing, safe food preparation, and access to clean water for prevention in HSCT recipients.

## Author contributions

**Project administration:** Yu Hu.

**Resources:** Han Yan, Yong You, Zhao-dong Zhong.

**Writing – original draft:** Shengling Ma.

**Writing – review & editing:** Wei Shi.
